# How do ageism, death anxiety and ageing anxiety among medical students and residents affect their attitude towards medical care for older patients: a systematic review

**DOI:** 10.1186/s12909-024-05147-1

**Published:** 2024-02-27

**Authors:** Emma J. Draper, Ariadne A. Meiboom, Nynke van Dijk, Johannes C. F. Ket, Rashmi A. Kusurkar, Martin Smalbrugge

**Affiliations:** 1 Department of Medicine for Older People, Amsterdam UMC Location Vrije Universiteit Amsterdam, Amsterdam, The Netherlands; 2 Amsterdam Public Health, Aging and Later Life, Amsterdam, The Netherlands; 3 Faculty of Health and Faculty of Sports and Nutrition, Center of Expertise Urban Vitality, Amsterdam University of Applied Sciences, Amsterdam, The Netherlands; 4 Department of General Practice, Amsterdam UMC Location University of Amsterdam, Amsterdam, The Netherlands; 5 Medical Library, University Library, Vrije Universiteit, Amsterdam, The Netherlands; 6 Department of Research in Education, Amsterdam UMC Location Vrije Universiteit Amsterdam, Amsterdam, The Netherlands; 7 LEARN! Research Institute for Learning and Education, Faculty of Psychology and Education, VU University Amsterdam, Amsterdam, The Netherlands; 8 Amsterdam Public Health, Quality of Care, Amsterdam, The Netherlands

**Keywords:** Medical education, Fear of death, Age discrimination, Geriatric medicine, Older adult, Perception on aging

## Abstract

**Background:**

Although the number of older patients requiring medical care is increasing, caring for older patients is often seen as unattractive by medical trainees (i.e., medical students, residents, interns, and fellows). Terror Management Theory states that people have a negative attitude towards older people, because they remind people of their own mortality. We hypothesize that ageism, death anxiety, and ageing anxiety among medical trainees negatively affect their attitude towards medical care for older patients. This review aimed to examine and generate an overview of available literature on the relationship between ageism, death anxiety, and ageing anxiety among medical trainees and their attitude towards medical care for older patients.

**Methods:**

A systematic review was performed with a review protocol based on the PRISMA Statement. PubMed, Ebsco/PsycInfo, Ebsco/ERIC and Embase were searched from inception to August 2022, using the following search terms, including their synonyms and closely related words: “medical trainees” AND “ageism” OR “death anxiety” OR “ageing anxiety” AND “(attitude AND older patient)”.

**Results:**

The search yielded 4072 different studies; 12 eligible studies (10 quantitative and 2 qualitative) were identified and synthesized using narrative synthesis. Findings suggest that a positive attitude towards older people was related to a positive attitude towards medical care for older patients among medical students. The available literature on the relationship between death anxiety and/or ageing anxiety and attitude towards medical care for older patients among medical trainees was limited and had a heterogeneity in focus, which hindered comparison of results.

**Conclusion:**

Our findings suggest that a positive attitude towards older people in general is related to a positive attitude towards medical care for older patients among medical students. Future research should focus on further exploring underlying mechanisms affecting the attitude towards medical care for older patients among medical trainees.

**Supplementary Information:**

The online version contains supplementary material available at 10.1186/s12909-024-05147-1.

## Introduction

As the ageing population is growing, the proportion of older people requiring medical care is increasing. Although physicians are more and more likely to work with older patients, there is little interest among physicians to provide medical care for older patients. Specializing in medical care for older patients, such as geriatric medicine or elderly care medicine (i.e., specialization for physicians working in nursing homes in the Netherlands), is consistently considered an unpopular career choice [1]. Also, physicians in general seem to have an ambivalent or negative view of medical care for older patients [2, 3].

Previous studies have identified factors that affect the attitude towards medical care for older patients among medical trainees (i.e., medical students, residents, interns, fellows). Characteristics of medical care for older patients, such as chronic, psychosocial or end-of-life care, are seen as unattractive [4, 5]. Also, lower professional status and lower financial reward of geriatric medicine could negatively influence students’ attitude towards medical care for older patients [5]. Meiboom et al. found that a lack of exposure to geriatric medicine during medical school resulted in a negative perception of this specialism [5]. However, another study found that the interest in becoming an elderly care physician did not increase after doing an elderly care medicine clerkship [6]. Although students did find various characteristics of elderly care medicine attractive, such as teamwork, communication and cognitive challenges, their interest in the specialism remained low.

This indicates a need to explore other factors or underlying mechanisms which may affect medical trainees’ attitude towards medical care for older patients. These other factors or underlying mechanisms could provide a focus for designing interventions aimed at fostering a more positive attitude during medical education. Ageism, which is prejudice, stereotyping and discrimination directed to individuals based on their age [7], could be an underlying mechanism affecting medical trainees’ attitude towards medical care for older patients. The phenomenon of ageism could be explained by Terror Management Theory [8]. This model posits that the confrontation with older people raises awareness about one’s own mortality, and subsequently increases death anxiety and ageing anxiety. Subconsciously people try to protect themselves from these unpleasant, frightening feelings by engaging defensive attitudes and behaviors towards older people [9]. These self-protective mechanisms could affect medical trainees’ attitude towards medical care for older patients.

Ageism, death anxiety and ageing anxiety negatively affect interest in caring for older patients in related professions. For instance, Mejia et al. showed a positive association between death anxiety and negative behaviors towards older people among psychology trainees [10]. Both factors were negatively associated with trainees’ willingness and desire to work with older people. Boswell found that greater ageing anxiety among psychology and prenursing undergraduate students was related to lower interest in working with older patients and ageist attitudes [11].

**Fig. 1:**
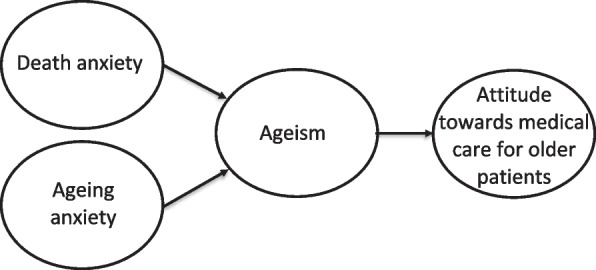
Hypothesized relationship between death anxiety, ageing anxiety, and ageism (i.e. underlying mechanisms) and attitude towards medical care for older patients

We hypothesize that ageism, death anxiety, and ageing anxiety among medical trainees negatively affect their attitude towards medical care for older patients (see Fig. 1). Therefore, this systematic review aimed to explore results of studies describing the relationship between ageism, death anxiety, and ageing anxiety among medical trainees and their attitude towards medical care for older patients. Findings may enhance our understanding of the underlying mechanisms that contribute to medical trainees’ attitude towards medical care for older patients, offer directions for medical education and, thereby, contribute to an increased willingness and enthusiasm for medical care for older patients among the physicians of the future.

## Methods

This systematic review was reported in accordance with the PRISMA Statement (www.prisma-statement.org). Our systematic review protocol is available on request.

### Search strategy

The search strategy was developed in collaboration with an information specialist (JCFK) and sharpened after a preliminary search. The databases PubMed, Embase, Ebsco/PsycInfo and Ebsco/ERIC databases were searched from inception up to August 2022. The following search terms were used as index terms or free-text words, including synonyms and closely related words: “medical trainees” AND “ageism” OR “death anxiety” OR “ageing anxiety” AND “(attitude AND older patient)”. The full search strategy can be found in Additional file 1. There were no language and date restrictions. Additional studies were identified through forward citation chaining (i.e., papers citing the included papers) and backward chaining (i.e., papers cited by the included papers) (date 16–11-2022).

### Ageism

In our preliminary search, we identified potentially relevant studies that defined ageism as a negative attitude towards older people. To provide a complete overview of the available literature on our research question, we expanded our focus from ageism to the entire spectrum of possible attitudes towards older people.

### Eligibility criteria

**Table:** 

*Criteria*	*Inclusion*	*Exclusion*
*Types of publication*	Empirical publications	Opinions, commentaries, conference abstracts, theses, books
*Population*	Medical trainees, i.e. medical students, residents, interns, fellows	> 25% Health care professionals or students in addition to medical trainees and not specifying outcomes for medical trainees
*Types of research*	Quantitative, qualitative, mixed methods	Case reports, (systematic) reviews
*Focus of article*	Relationship between ageism (or other attitudes towards older people), death anxiety and/or ageing anxiety, and attitude towards medical care for older patients (interest in geriatric medicine as a career is also regarded as positive attitude towards medical care for older patients)	-

### Study selection

After excluding duplicates, two authors (EJD and AAM) independently screened all titles and abstracts to identify articles possibly relevant for inclusion in the review. Titles and abstract retrieved by forward and backward citation chaining were screened independently by EJD and AAM or RAK. The full text of potentially eligible articles were reviewed independently by EJD and AAM. Agreement about eligibility was reached through discussion. In case of non-agreement, a third author (RAK) was consulted.

### Data extraction and synthesis

Data were extracted by EJD and checked by AAM, using an extraction form including: first author, year of publication, country, type of publication, description of the sample, description of underlying mechanism (i.e., attitude towards older people, death anxiety, or ageing anxiety) and description of attitude towards medical care for older patients (see Additional file 2). Agreement about data extraction was reached through discussion. In case of doubt a third author (RAK) was consulted. A narrative synthesis approach was adopted, based on description of the relevant findings in the quantitative and qualitative studies, and evaluation of the robustness of the findings in relation to the quality of the studies [12].

### Quality assessment

Two authors (EJD and AAM) independently assessed the methodological quality of the included studies. The Consolidated Criteria for Reporting Qualitative Studies (COREQ) [13] was used to assess the quality of qualitative studies on the following eight domains: personal characteristics, relationship with participants, theoretical framework, participant selection, setting, data collection, data analysis and reporting. Another rating form by Bland et al. [14] was used to assess the quality of quantitative studies on the following four domains: type of study, data source, theory or model based, and sample size.

Both rating instruments were used to score the methodological quality of the included articles and to calculate a total score, which was represented as a percentage of the total number of points applicable. Articles achieving a total score of 45% or higher were considered to be of good quality [14]. Although methodological quality of the studies was evaluated, studies were not excluded on this basis. We aimed to offer an overview of the entire available literature related to our topic of interest.

### Research team

EJD, AAM, ND, RAK and MS are researchers in medical education, AAM and MS are medical specialists in elderly care medicine and EJD, ND and RAK are trained as medical doctors. EJD and AAM, MS are academic teachers in medicine. JCFK is an information specialist.

## Results

**Fig. 2:**
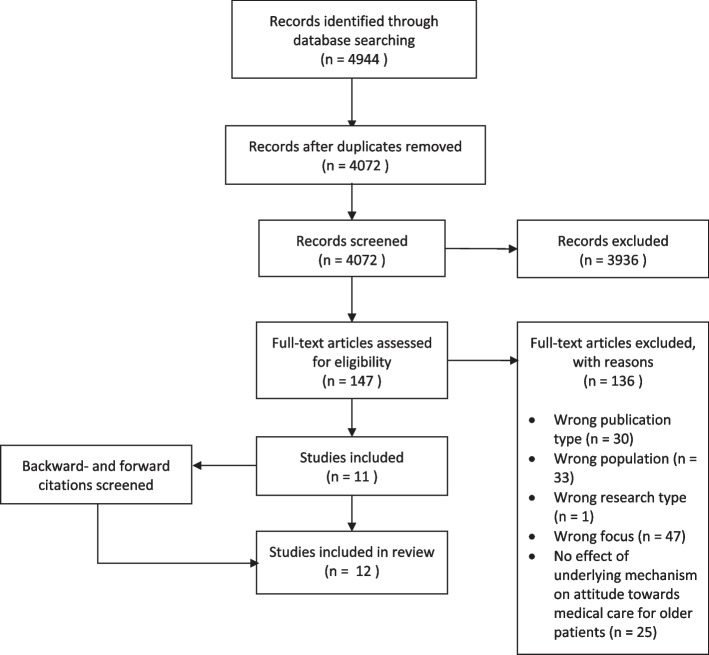
Flow diagram of literature search and study selection

The search yielded 4072 different articles: 147 were identified as relevant after initial screening of titles and abstracts and 11 articles were included after reviewing the full texts. Backward and forward citation chaining yielded one eligible study. See Fig. 2.

### Study characteristics

The review included a total of 12 studies conducted from 1981 – 2021 in the USA (*n* = 6), UK (*n* = 1), Singapore (*n* = 1), Malaysia (*n* = 1), Taiwan (*n* = 1), China (*n* = 1) and the Netherlands (*n* = 1). Out of these, 10 were quantitative and 2 were qualitative studies. Target populations were medical students (from different study years; *n* = 11) and residents (*n* = 1). The majority of the included articles, with the exception of two, were assessed to be of good quality (as described before). See Table 1 for a complete list of study characteristics and results.

**Table 1:** Characteristics and results of the included studies

First author (year)	Study aims	Design	Sample (participants (*N*), response rate, level, specialism)	Setting	Country	Measure of attitude towards death/ageing/older people	Measure of attitude towards medical care for older patients	Relevant findings	Quality assessment (percentage of total)
Chua [15]	To determine medical students’ attitudes towards older people and their willingness to consider a career in geriatric medicine	Quantitative; cross-sectional survey	244; RR 97,6%; Medical students (first year, prior to formal clinical exposure)	Medical school; Yong Loo Lin School of Medicine, National University of Singapore	Singapore	Attitudes towards older people using a modified UCLA-GAS (14 items)[27]	Willingness to consider geriatric medicine as a career choice (1 item)	Significant positive relationship between medical students’ attitudes towards older people and the willingness to consider geriatric medicine as a career (*r* = *0.48, p* < 0.001)	45%
Fitzgerald [16]	To examine the relationship between medical students’ attitudes toward older adults and their interest in geriatric medicine as a career	Quantitative; cross-sectional survey	171; RR 89%; Medical students (first year, entering medical school)	Medical school; University of Michigan	USA	Attitudes about older adults using UCLA-GAS (14 items)[27]	Interest in geriatric medicine as a career (1 item)	Significant positive relationship between medical students’ attitudes towards older adults and interest in a geriatric medicine career (β = 0.28, *t* = 0.28, *p* < 0.001)	49%
Hughes [20]	To evaluate the relationship between medical students’ attitudes toward older people and their willingness to consider a career in geriatric medicine, and the effects of a geriatric medicine training program	Quantitative; cross-sectional survey and one group pretest–posttest study	233; RR 99% and 58%; Medical students (first year before clinical exposure (*n* = 169), fourth year before and after geriatric medicine clinical training program (*n* = 70))	Medical school; University of Aberdeen	UK	Attitudes toward older people using a modified UCLA-GAS (14 items)[27]	Likelihood of considering a career in geriatric medicine (1 item)	Significant positive relationship between first year medical students’ attitude toward older people and their willingness to consider a career in geriatric medicine (*R*^*2*^ = 0.124, *p* < 0.001). No significant association between the post course rise of fourth year medical students’ willingness to consider a career in geriatric medicine and their attitude towards older people (*p* > 0.20)	43%
Meiboom [26]	To gain more insight in factors that have influenced postgraduate medical trainees in choosing elderly care medicine as a career during medical school	Qualitative; focus group discussions	34–35; RR unknown; Postgraduate medical trainees (elderly care medicine (*n* = 26–27) and gynecology (*n* = 8)	University medical center and a teaching hospital; VU University Medical Center and Saint Lucas Andreas Hospital, the Netherlands	The Netherlands	n.a	n.a	Some elderly care trainees were drawn to older patients because of their wisdom and stories. For some of them this played a role in choosing elderly care as a specialism, for others it did not play a role. Some trainees were scared by death, resulting in a negative perception of elderly care medicine during their medical education	63%
Merrill [21]	To investigate the reason some caregivers desire to avoid patients with terminal illnesses	Quantitative; cross-sectional survey	718; RR unknown; Caregivers (medical students (first and fourth year; *n* = 616), primary care physicians (*n* = 55), nurse students (undergraduate and graduate; *n* = 47))	Unknown, except for physicians; practicing in the community health clinics of Houston, Texas	USA	Thanatophobia using a rewritten Thanatophobia Scale (7 items)[33]	Self-esteem in caring for elderly patients using an unnamed scale (10 items)[34]	Significant negative relationship between fourth year medical students’ thanatophobia scores and self-esteem when treating elderly persons (*p* < 0.0001). Relationship between thanatophobia among first year students and their self-esteem when treating elderly persons was not reported	50%
Ng [28]	To analyze medical students’ attitude towards older adults and interest in geriatric medicine as a career before and after implementation of a course	Quantitative; pretest–posttest study	68; RR 71%; Medical students (third year, before and after Healthcare of Elderly course)	Medical school; Universiti Putra Malaysia	Malaysia	Attitude towards older adults using UCLA-GAS (14 items)[27]	Interest to pursue geriatric medicine as a career (number of items unknown)	No significant relationship between post course third year medical students’ attitude toward older adults and their interest in a future career in geriatric medicine (β(SE) = 0.38 (0.26), 95% CI = 0.14, 0.90, *p* = 0.152)	39%
Perrotta [17]	To examine the influence of medical students’ general attitudes toward the aged on their attitudes toward geriatric patients and geriatric medicine	Quantitative; cross-sectional survey	127; RR 100%; Medical students (first-year)	Medical school; attendees of the first-year student orientation program at the State University of New York at Buffalo School of Medicine	USA	Attitudes toward the aged using Kogan scale (34 items)[29]	Attitudes toward geriatric patients and geriatric medicine using a questionnaire containing items developed by various authors (8 items; 5 subscales, i.e. Characteristics of Elderly Patients, Choice of Patient Population (preference for younger or older patients and interest in geriatric medicine), The Chance of Successfully Treating the Aged, Social and Emotional Characteristics of Elderly Patients, Amount that Elderly Patients Contribute to the Treatment Process)[35–37]	Significant positive relationship between first year medical students’ attitude toward the aged and the subscale characteristics of elderly patients (*r* = 0.27, *p* = 0.006). No significant relationship between first year medical students’ attitude towards the aged and their attitudes towards geriatric patients and geriatric medicine (and other subscales of the questionnaire)	50%
Ruiz [22]	To determine the relationship between medical students’ explicit and implicit anti-aging bias and their intent to treat older patients	Quantitative; cross-sectional survey	103; RR 14%; Medical students (first year (*n* = 33), second year (*n* = 35), third year (*n* = 22), fourth year (*n* = 12))	Medical school; attendees of a Liaison Committee on Medical Education (LCME) accredited medical school in the USA	USA	Attitudes toward the elderly using Fraboni Scale of Ageism (29 items; explicit attitude)[31] and Implicit Association Test (pairing 10 pleasant and unpleasant words with pictures of old and young men; implicit attitude)[39]	Intent to practice with older patients using Intended Practice Patterns with Older Patients (10 items), adapted from unnamed questionnaire[38]	Significant positive relationship between medical students’ explicit attitude toward the elderly and their intent(ion) to practice with older patients (*r* = 0.38, *p* < 0.001). No significant relationship between their implicit attitude toward the elderly and intent(ion) to practice with older patients	46%
Schigelone [18]	To examine medical students’ attitudes, experiences, beliefs about older adults, and their fears about aging and death, that are relevant to their pursuit of geriatrics	Qualitative; semi-structured interviews	20; RR 53%; Medical students (first year), sample contained students that were moderately interested (*n* = 10) or not interested (*n* = 10) in geriatric medicine as a career	Medical school; situated Midwestern	USA	n.a	n.a	Students who were moderately interested in geriatrics expressed much more fear about aging and death than those students who were not interested in geriatrics. Those who were afraid expressed more fears about the death of others close to them than about their own death. This concern about death extended to their patients as well. Their fears of aging applied to both the aging of others and of themselves	50%
Wilderom [19]	To examine medical students’ attitudinal correlates to specialize in geriatric medicine	Quantitative; cross-sectional survey	663; RR 82%; Medical students (first year, entering medical school; in 6 consecutive years)	Medical school; a large northeastern American state university school of medicine	USA	Attitude toward the aged in general using Kogan scale (34 items)[29]	Interest in providing medical care to elderly patients and in specializing in geriatric medicine (number of items unknown).Perception of the elderly as patients (number of items unknown)	Significant positive relationship between first year medical students’ attitude toward the aged and their perception of the elderly as patients. Attitude toward the aged explained 10% of the variation in perception of the elderly as patients. Significant positive relationship between first year medical students’ attitude toward the aged and their interest in specializing in geriatric medicine. Attitude toward the aged explained 2% of the variation in their interest in specializing in geriatrics	57%
Yao [23]	To explore medical students’ willingness to work with older persons and the associated factors	Quantitative; cross-sectional survey	580; RR 72,5%; Medical students (first year (*n* = 85), second year (*n* = 146), third year (*n* = 199), fourth year (*n* = 150)	Medical school; 6 or 7 Medical universities situated in Taiwan	Taiwan	Attitudes towards older adults using (Polizzi’s refined version of the) Aging Semantic Differential (24 items)[30]	Willingness to take care of older adults using Willingness towards the Elderly Care Scale (15 items)	Significant positive relationship between medical students’ attitudes towards older adults and their willingness to care for older adults (*r* = 0,313, *p* < 0.000)	51%
Zhao [24]	To examine the relationship between attitude toward older adults and willingness to consider a career in geriatric medicine	Quantitative; cross-sectional survey	1022; RR 96%; Medical students (first year (*n* = 212), second year (*n* = 273), third year (*n* = 300), fourth year (*n* = 172), fifth year (*n* = 65)	Medical school; Huanzhong University of Science and Technology and Shanxi Medical University	China	Attitude towards older adults using Fraboni Scale of Ageism (29 items)[31]	Willingness to consider a career in geriatric medicine after graduation (1 item)	Significant positive relationship between medical students’ attitude towards older adults and their willingness to consider a career in geriatric medicine after graduation (*t* = 4.281, *p* < 0,001, Cohen’s *d* = 0.268)	57%

#### Participants

Among the included articles, five articles merely included first year medical students without clinical experience [15–19], five articles included a mix of medical students with or without clinical experience [20–24], one article included third year medical students with clinical experience [25] and one article included postgraduate medical trainees (i.e., residents and junior doctors) [26]. This distinction is of importance, because first year medical students without clinical experience may lack or have an inaccurate understanding of what medical care for older patients entails.

#### Measurement of attitude towards older people

Included quantitative studies assessed the attitudes towards older people using different questionnaires. The University of California at Los Angeles Geriatric Attitude Scale (UCLA-GAS) [27] was used in four of the included studies [15, 16, 20, 28]. The UCLA-GAS assesses attitudes toward older people and geriatric patient care in four dimensions: i) perceived social value of older people (e.g., “In general, old people act too slow for modern society”), ii) medical care provided to geriatric patients (e.g., “Taking a medical history from elderly patients is frequently an ordeal”), iii) compassion toward older people (e.g., “I tend to pay more attention and have more sympathy toward my elderly patients than my younger patients”), iv) distribution of societal resources for older people (e.g., “Old people in general do not contribute much to society”). The outcome of the UCLA-GAS was defined as positive or negative attitude towards older people, in which a high score represented a positive attitude.

Perrotta et al. and Wilderom et al. [17, 19] used the Kogan scale [29] to assess the attitude towards older people. Yao et al. [23] used (a refined version of) the Aging Semantic Differential scale [30]. Ruiz et al. and Zhao et al. [22, 24] used the Fraboni Scale of Ageism [31] to measure the attitude towards older people among medical students. In addition, Ruiz et al. also used an Implicit Association Test [32] to measure their implicit attitude towards older people.

The qualitative study of Meiboom et al. reported on being drawn to older patients because of their wisdom and stories [26].

#### Measurement of death anxiety and ageing anxiety

Merrill et al. [21] used the Thanatophobia Scale [33] to assess attitude towards caring for dying patients.

The qualitative study of Meiboom et al. reported on being scared by death (while providing medical care for older patients) [26]. The qualitative study of Schigelone and Ingersoll-Dayton asked participants about their fears about ageing and death [18].

#### Measurement of attitude towards medical care for older patients

The included quantitative studies examining attitude towards medical care for older patients among medical trainees had a heterogeneity in focus. Three dimensions of the attitude towards medical for older patients could be recognized, namely i) attitude towards older patients, ii) attitude towards providing medical care for older patients, and iii) interest in pursuing geriatric medicine as a future career. Two articles measured the attitude towards older patients [17, 19]. Three included articles measured the attitude towards providing medical care to older patients using different scales [21–23]. Last, seven articles measured the interest in geriatric medicine as a future career among medical trainees, which we interpreted as a positive attitude towards medical care for older patients [15–17, 19, 20, 24, 28]. These articles used a single question (*n* = 4), a scale (*n* = 1) or an unknown number of items (*n* = 2).

The qualitative study of Meiboom et al. asked medical trainees about factors that have influenced their career choices [26]. The qualitative study of Schigelone and Ingersoll-Dayton asked medical students about the underlying reasons for their interest, or the lack thereof, in geriatric medicine [18].

### Relationship between attitude towards older people, death anxiety and ageing anxiety and attitude towards medical care for older patients

No articles were found which describe the relationship between all hypothesized underlying mechanisms (i.e., attitude towards older people, death anxiety, and ageing anxiety) among medical trainees and their attitude towards medical care for older patients. Subsequently, we will discuss the relationships between separate underlying mechanisms and the attitude towards medical care for older patients.

### Relationship between attitude towards older people and attitude towards medical care for older patients

The relationship between attitude towards older people and attitude towards medical care for older patients was investigated in nine included studies, whereof eight were quantitative and one was qualitative. The majority of our findings described that a more positive attitude towards older people among medical students was related to a more positive attitude towards medical care for older patients. Four included articles found that first year medical students with a more positive attitude towards older people had more interest in specializing in geriatric medicine [15, 16, 19, 20]. However, Wilderom et al. [19] found that first year medical students’ attitude towards older people only explained 2% of the variation in interest in geriatric specialization. Wilderom et al. [19] also found that first year medical students with a more positive attitude towards older people had a more positive perception of older patients. Their attitude towards older people explained 10% of their variation in their perception of older patients. Additionally, Perrotta et al. [17], who used a cohort of students that was also included in the much larger sample of Wilderom et al. [19], found no significant relationship between the attitude towards older people among first year medical students and their attitudes towards geriatric patients and geriatric care.

Ruiz et al. [22] found that first to fourth year medical students with a more positive explicit attitude towards older people have higher intention to provide medical care to older patients after finishing medical school. However, their implicit (i.e. subconscious) attitude towards older people was not significantly related to their intention to provide medical care to older patients after finishing medical school. Yao et al. [23] found that first to fourth year medical students with a more positive attitude towards older adults were more willing to provide medical care for older patients. Zhao et al. [24], who had the largest sample size (*n* = 1022) among the included studies, found that first to fifth year medical students with a more positive attitude towards older people had more interest in specializing in geriatric medicine. Ng et al. [28] implemented an ageing and geriatric medicine course for third year medical students. No significant post course relationship was found between attitude towards older people and interest in specializing in geriatric medicine among third year medical students. Next to first year students, Hughes et al. [20] also examined fourth year medical students’ attitude towards older people and their interest in specializing in geriatric medicine before and after a clinical geriatric medicine teaching program. No significant relationship was found between their attitude towards older people and post course rise in interest in specializing in geriatric medicine. Both the studies of Ng et al. [28] and Hughes et al. scored poor on methodological quality. In a qualitative study, Meiboom et al. [26] found that some elderly care medicine trainees were drawn to older patients because of their wisdom and stories. For some this played a role in choosing to specialize in elderly care medicine, for others it did not.

Overall, nine studies found a significant positive relationship between attitude towards older people and attitude towards medical care for older patients, which aligned with our hypothesis [15–17, 20, 22–24], and in five studies this was inconclusive [17, 20, 22, 26, 28].

### Relationship between death anxiety and attitude towards medical care for older patients

The relationship between death anxiety among medical trainees and their attitude towards medical care for older patients was examined in three included articles, of which two were qualitative [18, 26] and one was quantitative [21]. On one hand we found, in the quantitative study of Merrill et al. [21], that higher death anxiety among fourth year medical students was a predictor of lower self-esteem when treating older patients. Meiboom et al. [26] found in focus groups that some medical trainees were scared by death, resulting in a negative perception of elderly care medicine during their medical education. On the other hand, in the qualitative study of Schigelone and Ingersoll-Dayton [18] first year medical students were asked if they were interested in specializing in geriatric medicine. Students who were moderately interested in geriatric medicine expressed much more fear about death than students who were not interested in geriatric medicine. Those who were afraid expressed more fears about the death of others close to them than about their own death. Some students that were not interested in geriatric medicine expressed that losing a younger patient would be more difficult than losing an older one.

Overall, three included studies examined the relationship between death anxiety and attitude towards medical care for older patients. In two studies the findings aligned with our hypothesis [21, 26], and in one study the findings contradicted our hypothesis [18].

### Relationship between ageing anxiety and attitude towards medical care for older patients

Schigelone and Ingersoll-Dayton [18] also studied the relationship between ageing anxiety and attitude towards medical care for older patients among first year medical students. This qualitative study, which is the only one included in this review addressing this relationship, found that students expressing more fear about ageing were more interested in specializing in geriatric medicine. Their fears of ageing applied to both the ageing of others and of themselves. Losing their cognitive functioning was the greatest fear for many students, they expressed this being completely out of own control.

Thus, the included study about the relationship between ageing anxiety and attitude towards medical care for older patients contradicted our hypothesis [18].

## Discussion

Following the need for more insight into the underlying mechanisms that may affect medical trainees’ attitude towards medical care for older patients, the purpose of this systematic review and narrative synthesis was to explore and provide an overview of the available literature on the relationship between attitude towards older people, death anxiety, and ageing anxiety among medical trainees and their attitude towards medical care for older patients.

No evidence was found to support our hypothesized relationship between all underlying mechanisms (i.e., attitude towards older people, death anxiety and ageing anxiety) among medical trainees and their attitude towards medical care for older patients (Fig. 1).

The available literature reporting about the relationships between death anxiety and/or ageing anxiety among medical trainees and their attitude towards medical care for older patients was limited and had a heterogeneity in focus and methodology, which hindered comparison of the results. Contrary to our hypothesis, one qualitative study included in this review found that more interest in geriatric medicine was related to more fears about death and ageing among first year medical students [18]. Medical students seemed to be more attracted to fields where they had the greatest fears. Possibly these students try to solve or seek control over situations they are most afraid of themselves.

The findings do suggest, that a more positive attitude towards older people is related to a more positive attitude towards medical care for older patients among medical students. This conclusion was drawn in studies that were assessed to have good methodological quality. While the findings support our hypothesis, it is important to note the modest strength of this relationship, which suggests that there may be other potentially more influential factors or underlying mechanisms influencing medical students’ attitude towards medical care for older patients. Hughes et al. described that the interest in specializing in geriatric medicine among medical students significantly raised after a clinical geriatric medicine teaching program, although their attitude towards older people did not rise [20]. This finding suggests that an enhanced sense of competence gained through education focused on geriatric medicine may subsequently contribute to a more positive attitude towards medical care for older patients. This would warrant more attention to geriatric medicine in medical education, in order to improve the attitude towards medical care for older patients.

Also, the robustness of the findings may be compromised as the instruments used in the studies included in our review measuring the attitude towards older people among medical students exhibit certain limitations. Despite being the most frequently used instrument, the UCLA-GAS, shows some issues. Previous research found that the internal consistency of the measurement with UCLA-GAS is unacceptable, revealing reliability problems [40].

Although the scale was frequently used to assess the attitude towards older people, it was originally developed to assess attitudes towards older people and caring for older patients [27]. The scale has four subscales, of which one consists of statements about the attitude towards providing care to geriatric patients. This is especially problematic for our review, because we were investigating both these (or closely related) concepts (i.e., attitude towards older people and attitude towards medical care for older patients).

Additionally, most studies investigated the underlying mechanisms through use of self-reported questionnaires, which can suffer from several kinds of bias. Ageism and other attitudes towards older people are closely related to prejudice, stereotyping and discrimination towards individuals based on their age, which are sensitive to social desirability bias. Previous studies have investigated prejudice using dual process models that include explicit and implicit levels of prejudice [41]. Explicit biases involve deliberative, controlled processes, whereas implicit biases involve automatic, mainly unconscious processes. Death anxiety and ageing anxiety are possibly even more difficult to self-report, because they are mostly unconscious processes. At a conscious level, most people report not to be afraid of death, whereas beneath their consciousness they feel averse to death [42].

Most studies in our review investigated first year medical students without any clinical experience and measured their interest in specializing in geriatric medicine after graduation (which we interpret as a positive attitude towards medical care for older patients). It is unclear which expectations first year students have of medical care for older patients or geriatric medicine. In a qualitative study included in our review, the general perception of geriatric medicine among first year medical students was that they would be spending most of the day chatting with older people [18]. This calls for an earlier introduction of older patients to medical students, so they can experience that medical care for older patients entails much more than chatting with older people.

### Strengths and limitations

The strength of this systematic review is the extensive search strategy and including forwards and backwards citation chaining search, after which we were able to identify a critical gap in the current knowledge. A limitation is our decision to not select studies on methodological quality, which could generate bias in our systematic review. However, due to the small number of studies, the effect of this decision was difficult to establish. Also, the terminology that was used for the constructs of our interest, especially the attitude towards older people and the attitude towards medical care for older patients, varied widely. This made it difficult to compare the results of the included studies.

### Future research

Future research should focus on further exploring medical trainees’ attitude towards medical care for older patients and the underlying mechanisms affecting this attitude. More senior medical students with experience providing medical care for older patients could be more suitable than first year medical students without these experiences, which were frequently used as participants in the included studies. Their experiences, and possible reflection on these experiences, could have attributed to forming their attitude towards medical care for older patients. More alternative methods, such as rich pictures, observations or ethnography could be effective ways of mapping out their attitudes and underlying mechanisms affecting their attitudes. Deeper insight into medical trainees’ attitude towards medical care for older patients could provide directions for medical education and, thereby, contribute to generating more enthusiasm and willingness for providing medical care for older patients.

## Conclusion

Findings suggest that a more positive attitude towards older people is related to a more positive attitude towards medical care for older patients among medical students. There was insufficient evidence to support our hypothesized relationship between all underlying mechanisms (i.e., attitude towards older people, death anxiety and ageing anxiety) among medical trainees and their attitude towards medical care for older patients (Fig. 1). Future research should focus on gaining a deeper understanding of the attitude towards medical care for older patients through qualitative studies.

### Supplementary Information

**Additional file 1.** Search strategy.

**Additional file 2.** Extraction form.

## Data Availability

All data generated or analyzed during this study are included in this published article and its additional information files.

## Abbreviation

UCLA-GAS: University of California at Los Angeles- Geriatric Attitude Scale

## References

[1] Album D, Westin S (2008). Do diseases have a prestige hierarchy? A survey among physicians and medical students. Soc Sci Med.

[2] Higashi RT, Tillack AA, Steinman M, Harper M, Johnston CB (2012). Elder care as "frustrating" and "boring": understanding the persistence of negative attitudes toward older patients among physicians-in-training. J Aging Stud.

[3] Meiboom, Diedrich C, De Vries H, Hertogh C, Scheele F. The Hidden Curriculum of the Medical Care for Elderly Patients in Medical Education: A Qualitative Study. Gerontology & Geriatrics Education. 2015;36(1):30–44. 10.1080/02701960.2014.966902 25288267

[4] Bagri AS, Tiberius R (2010). Medical student perspectives on geriatrics and geriatric education. J Am Geriatr Soc.

[5] Meiboom, de Vries H, Hertogh C, Scheele F. Why medical students do not choose a career in geriatrics: a systematic review. Bmc Medical Education. 2015;15. 10.1186/s12909-015-0384-4 PMC4470031 26043772

[6] Meiboom, de Vries H, Soethout MBM, Hertogh CMPM, Scheele F. Een carrière als specialist ouderengeneeskunde; iets voor de huidige geneeskundestudent? Belangstelling geneeskundestudenten voor ouderengeneeskunde [A career in elderly care medicine; an option for today’s medical student? Medical students’ interest in elderly care medicine]. Tijdschrift voor Gerontologie en Geriatrie. 2018;49(4):139–46. 10.1007/s12439-018-0255-7 30003475

[7] Wilkinson JA, Ferraro KF. Thirty years of ageism research. 2002.

[8] Greenberg J, Pyszczynski T, Solomon S (1986). The causes and consequences of a need for self-esteem: A terror management theory.

[9] Martens A, Goldenberg JL, Greenberg J (2005). A terror management perspective on ageism. J Soc Issues.

[10] Mejia M, Hyman SM, Behbahani S, Farrell-Turner K (2018). Death anxiety and ageist attitudes are related to trainees' interest in working with older adults. Gerontol Geriatr Educ.

[11] Boswell SS (2012). 'Old people are cranky': Helping professional trainees' knowledge, attitudes, aging anxiety, and interest in working with older adults. Educ Gerontol.

[12] Popay J, Roberts H, Sowden A, Petticrew M, Arai L, Rodgers M (2006). Guidance on the conduct of narrative synthesis in systematic reviews. A product from the ESRC methods programme Version.

[13] Tong A, Sainsbury P, Craig J (2007). Consolidated criteria for reporting qualitative research (COREQ): a 32-item checklist for interviews and focus groups. Int J Qual Health Care.

[14] Bland CJ, Meurer LN, Maldonado G. A systematic approach to conducting a non-statistical meta-analysis of research literature. Academic Medicine. 1995. 10.1097/00001888-199507000-00014 7612129

[15] Chua MP, Soiza RL (2009). Attitudes of first year medical students in Singapore towards older people and willingness to consider a career in geriatric medicine. Ann Acad Med Singapore.

[16] Fitzgerald JT, Wray LA, Halter JB, Williams BC, Supiano MA (2003). Relating Medical Students' Knowledge, Attitudes, and Experience to an Interest in Geriatric Medicine. Gerontologist.

[17] Perrotta P, Perkins D, Schimpfhauser F, Calkins E (1981). Medical Student Attitudes toward Geriatric Medicine and Patients. J Med Educ.

[18] Schigelone AS, Ingersoll-Dayton B (2004). Some of my Best Friends are Old: A Qualitative Exploration of Medical Students' Interest in Geriatrics. Educ Gerontol.

[19] Wilderom CPM, Press EG, Perkins DV, Tebes JA, Nichols L, Calkins E (1990). Correlates of entering medical students' attitudes toward geriatrics. Educ Gerontol.

[20] Hughes NJ, Soiza RL, Chua M, Hoyle GE, MacDonald A, Primrose WR, Seymour DG (2008). Medical student attitudes toward older people and willingness to consider a career in geriatric medicine. J Am Geriatr Soc.

[21] Merrill, Lorimor R, Thornby J, Woods A. Caring for terminally ill persons: comparative analysis of attitudes (thanatophobia) of practicing physicians, student nurses, and medical students. Psychol Rep. 1998;83(1):123–8. 10.2466/pr0.1998.83.1.123 9775670

[22] Ruiz JG, Andrade AD, Anam R, Taldone S, Karanam C, Hogue C, Mintzer MJ (2015). Group-based differences in anti-aging bias among medical students. Gerontol Geriatr Educ.

[23] Yao C-T, Lin P-E, Huang I-C (2021). Cross-sectional assessment of medical students willingness to care toward older people in Taiwan. Educ Gerontol.

[24] Zhao H, Wu B, Shi J, Reifsnider E, Fan J, Li J, Mao J (2020). Chinese Medical Students' Attitudes toward Older Adults and Willingness To Consider a Career in Geriatric Medicine: A Cross-Sectional Survey. Teach Learn Med.

[25] Green SK, Keith KJ, Pawlson LG (1983). Medical students' attitudes toward the elderly. J Am Geriatr Soc.

[26] Meiboom, de Vries H, Hesselink BA, Hertogh CM, Scheele F. Gegrepen door de ouderengeneeskunde, maar niet tijdens de studie geneeskunde! Keuze voor de opleiding tot specialist ouderengeneeskunde [Drawn towards a career in elderly care medicine, but not till after medical school. Elderly care medicine as a career choice]. Tijdschr Gerontol Geriatr. 2014;45(1):10–8. 10.1007/s12439-013-0056-y 24399288

[27] Reuben DB, Lee M, Davis JW, Eslami MS, Osterweil DG, Melchiore S, Weintraub NT (1998). Development and validation of a geriatrics attitudes scale for primary care residents. J Am Geriatr Soc.

[28] Ng ZL, Mat Din H, Zakaria NF, Inche Mat LN, Wan Zukiman WZH, Md Shah A (2021). Implementation of a Healthcare of Elderly Course With Multi-Professional Teachers for Undergraduate Medical Students in a Public University in Malaysia-A Quasi-Experimental Pre and Post Study. Front Public Health.

[29] Kogan N (1961). Attitudes toward old people: the development of a scale and an examination of correlates. J Abnorm Soc Psychol.

[30] Rosencranz HA, McNevin TE (1969). A factor analysis of attitudes toward the aged. Gerontologist.

[31] Fraboni M, Saltstone R, Hughes S (1990). The Fraboni Scale of Ageism (Fsa) - an Attempt at a More Precise Measure of Ageism. Canadian Journal on Aging-Revue Canadienne Du Vieillissement.

[32] Greenwald AG, McGhee DE, Schwartz JL (1998). Measuring individual differences in implicit cognition: the implicit association test. J Pers Soc Psychol.

[33] Merrill, Laux L, Thornby JI. AIDS and student attitudes. South Med J. 1989;82(4):426–32. 10.1097/00007611-198904000-00006 2705068

[34] Merrill JM, Lorimor RJ, Thornby JI (1996). Woods A. Self-esteem and caregivers' attitudes toward elderly persons. Psychological Reports.

[35] Spence DL, Feigenbaum EM, Fitzgerald F, Roth J (1968). Medical student attitudes toward the geriatric patient. J Am Geriatr Soc.

[36] Gale J, Livesley B (1974). Attitudes towards geriatrics: a report of the King's survey. Age Ageing.

[37] Cicchetti DV, Fletcher CR, Lerner E, Coleman JV (1973). Effects of a social medicine course on the attitudes of medical students toward the elderly: A controlled study. J Gerontol.

[38] Helton MR, Pathman DE (2008). Caring for older patients: current attitudes and future plans of family medicine residents. Fam Med.

[39] Greenwald AG, McGhee DE, Schwartz JL (1998). Measuring individual differences in implicit cognition: the implicit association test. J Pers Soc Psychol.

[40] Stewart TJ, Roberts E, Eleazer P, Boland R, Wieland D (2006). Reliability and Validity Issues for Two Common Measures of Medical Students' Attitudes Toward Older Adults. Educ Gerontol.

[41] Devine PG (1989). Stereotypes and Prejudice - Their Automatic and Controlled Components. J Pers Soc Psychol.

[42] Feifel H, Branscomb AB (1973). Who's afraid of death?. J Abnorm Psychol.

