# Supplementary material for: How do ageism, death anxiety and ageing anxiety among medical students and residents affect their attitude towards medical care for older patients: a systematic review
Source: BMC Med Educ. 2024 Feb 27;24:199. doi: 10.1186/s12909-024-05147-1 (PMC10900813; doi:10.1186/s12909-024-05147-1)
Supplement: Supplementary file 1 — Additional file 1. Search strategy. [file 12909_2024_5147_MOESM1_ESM.docx]

**Appendix 1: Search strategy**

*Pubmed 25 August 2022*

1 AND (2 OR 3 OR 4) AND 5

1. ("Students, Medical"[Mesh] OR "medical student*"[tiab] OR "medicine student*"[tiab] OR "intern"[tiab] OR "interns"[tiab] OR "internship*"[tiab] OR "undergraduate*"[tiab] OR "trainee*"[tiab] OR "resident"[tiab] OR "residents"[tiab] OR "residenc*"[tiab] OR "clerk"[tiab] OR "clerks"[tiab] OR "clerkship*"[tiab] OR "fellow"[tiab] OR "fellows"[tiab] OR "fellowship*"[tiab] OR "rotation*"[tiab] OR "physicians in training"[tiab] OR "physician in training"[tiab] OR "future physician*"[tiab])

AND

2. ("Attitude to death"[Mesh] OR "attitude to death"[tiab] OR "attitudes to death"[tiab] OR thanatophobi*[tiab] OR "death anxiet*"[tiab] OR "fear of death"[tiab] OR "fear of dying"[tiab] OR "fearing death"[tiab] OR "death attitude*"[tiab] OR "death avoid*"[tiab] OR "death distress*"[tiab] OR ((death[tiab] OR mortal*[tiab]) AND salience[tiab]) OR "death orientation*"[tiab] OR "own death"[tiab] OR "own mortal*"[tiab] OR ("existential*"[tiab] AND ("vulnerab*"[tiab] OR "anxiet*"[tiab] OR "suffering*"[tiab])) OR "death aware*"[tiab] OR "afraid of death"[tiab] OR "death thought*"[tiab] OR "death acceptance"[tiab] OR "accepting death"[tiab] OR "denial of death"[tiab] OR "facing death"[tiab] OR "anxieties about death"[tiab] OR "anxiety about death"[tiab] OR "anxiety toward death"[tiab] OR "anxieties toward death"[tiab] OR "attitude toward death"[tiab] OR "attitude towards death"[tiab] OR "attitudes toward death"[tiab] OR "attitudes towards death"[tiab])

OR

3. ("Ageism"[Mesh] OR "ageism*"[tiab] OR "age discriminati*"[tiab] OR "ageist*"[tiab] OR "agist"[tiab] OR "age bias*"[tiab] OR "aging bias*"[tiab] OR "age based discriminati*"[tiab] OR "age stereotype*"[tiab] OR "age prejudice"[tiab] OR "age stigma"[tiab] OR "age related bias"[tiab] OR "age related stigma"[tiab] OR "age related discrimination"[tiab] OR (("Attitude"[Mesh] OR "attitude*"[tiab] OR "perception*"[tiab] OR "perceive*"[tiab] OR "belief*"[tiab] OR "believe*"[tiab] OR "preconcepti*"[tiab] OR "stereotyp*"[tiab] OR "bias"[tiab] OR "discriminat*"[tiab] OR "stigma*"[tiab] OR "prejudic*"[tiab]) AND ("Aged"[Mesh] OR "Aged, 80 and over"[Mesh] OR "Frail Elderly"[Mesh] OR "elder*"[tw] OR "eldest"[tw] OR "old age*"[tw] OR "oldest old*"[tw] OR "senior*"[tw] OR "senium"[tw] OR "very old*"[tw] OR "septuagenarian*"[tw] OR "octagenarian*"[tw] OR "octogenarian*"[tw] OR "nonagenarian*"[tw] OR "centarian*"[tw] OR "centenarian*"[tw] OR "supercentenarian*"[tw] OR "older people"[tw] OR "old people"[tiab] OR "older subject*"[tw] OR "older age*"[tw] OR "old age"[tiab] OR "older adult*"[tw] OR "older man"[tw] OR "older men"[tw] OR "older male*"[tw] OR "older woman"[tw] OR "older women"[tw] OR "older female*"[tw] OR "older population*"[tw] OR "older person*"[tw] OR "ageing people*"[tiab] OR "aging people*"[tiab] OR "ageing person*"[tiab] OR "aging person*"[tiab] OR "older individual*"[tiab]))

OR

4. (“Attitude”[Mesh] OR "attitude*"[tiab] OR "perception*"[tiab] OR "perceiv*"[tiab] OR "experience*"[tiab]) AND ("personal aging*"[tiab] OR "own aging*"[tiab] OR "personal ageing*"[tiab] OR "own ageing*"[tiab] OR "attitude toward aging"[tiab] OR "attitudes toward aging"[tiab] OR "attitude toward ageing"[tiab] OR "attitude toward aging"[tiab] OR "attitudes to ageing"[tiab] OR "attitudes to aging"[tiab] OR "attitudes about ageing"[tiab] OR "attitudes about aging"[tiab] OR "aging expectations"[tiab] OR "expectations of ageing"[tiab] OR "expectations of aging"[tiab] OR "expectations about ageing"[tiab] OR "expectations about aging"[tiab] OR "perceptions of aging"[tiab] OR "perceptions of ageing"[tiab] OR "perceptions about aging"[tiab] OR "perceptions about ageing"[tiab] OR "fear of aging"[tiab] OR "fear of ageing"[tiab] OR "aging anxiet*"[tiab] OR "ageing anxiet*"[tiab])

AND

5. ("Attitude"[Mesh] OR "attitude*"[tiab] OR "perception*"[tiab] OR "perceiv*"[tiab] OR "experienc*"[tiab] OR "career*"[tiab] OR "interest*"[tiab]) AND ("geriatri*"[tiab] OR "Geriatrics"[Mesh] OR "older patient*"[tiab] OR "ageing patient*"[tiab] OR "aging patient*"[tiab] OR "aged care"[tiab] OR "care for the elderly"[tiab] OR "elderly care"[tiab] OR "eldercare"[tiab] OR "elder care"[tiab] OR "elderly patient*"[tiab] OR "elder patient*"[tiab])

*Embase.com 25 August 2022*

1 AND (2 OR 3 OR 4) AND 5

1. ('medical student'/de OR 'resident'/exp OR ‘medical student*’:ti,ab,kw OR ‘medicine student*’:ti,ab,kw OR intern:ti,ab,kw OR interns:ti,ab,kw OR internship*:ti,ab,kw OR undergraduate*:ti,ab,kw OR trainee*:ti,ab,kw OR resident:ti,ab,kw OR residents:ti,ab,kw OR residenc*:ti,ab,kw OR clerk:ti,ab,kw OR clerks:ti,ab,kw OR clerkship*:ti,ab,kw OR fellow:ti,ab,kw OR fellows:ti,ab,kw OR fellowship*:ti,ab,kw OR rotation*:ti,ab,kw OR ‘physicians in training’:ti,ab,kw OR ‘physician in training’:ti,ab,kw OR ‘future physician*’:ti,ab,kw)

2. ('attitude to death'/exp OR ‘death anxiety’/exp OR 'Death Anxiety Scale'/exp OR 'death education'/exp OR ‘attitude to death’:ti,ab,kw OR ‘attitudes to death’:ti,ab,kw OR thanatophobi*:ti,ab,kw OR ‘death anxiet*’:ti,ab,kw OR ‘fear of death’:ti,ab,kw OR ‘fear of dying’:ti,ab,kw OR ‘fearing death’:ti,ab,kw OR ‘death attitude*’:ti,ab,kw OR ‘death avoid*’:ti,ab,kw OR ‘death distress*’:ti,ab,kw OR ((death:ti,ab,kw OR mortal*:ti,ab,kw) AND salience:ti,ab,kw) OR ‘death orientation*’:ti,ab,kw OR ‘own death’:ti,ab,kw OR ‘own mortal*’:ti,ab,kw OR ((‘existence’/exp OR existential*:ti,ab,kw) AND (vulnerab*:ti,ab,kw OR anxiet*:ti,ab,kw OR suffering*:ti,ab,kw)) OR ‘death aware*’:ti,ab,kw OR ‘afraid of death’:ti,ab,kw OR ‘fear of dying’:ti,ab,kw OR ‘death thought*’:ti,ab,kw OR ‘death acceptance’:ti,ab,kw OR ‘accepting death’:ti,ab,kw OR ‘denial of death’:ti,ab,kw OR ‘facing death’:ti,ab,kw OR ‘anxieties about death’:ti,ab,kw OR ‘anxiety about death’:ti,ab,kw OR ‘anxiety toward death’:ti,ab,kw OR ‘anxieties toward death’:ti,ab,kw OR (('attitude'/de OR attitude*:ti,ab,kw) AND ('dying'/exp OR 'death'/de OR 'mortality'/de OR dying:ti,ab,kw OR death*:ti,ab,kw OR mortal*:ti,ab,kw)))

3. ('ageism'/exp OR ageism*:ti,ab,kw OR age discriminati*:ti,ab,kw OR ageist*:ti,ab,kw OR agist:ti,ab,kw OR ‘age bias*’:ti,ab,kw OR ‘aging bias*’:ti,ab,kw OR ‘age based discriminati*’:ti,ab,kw OR ‘age stereotype*’:ti,ab,kw OR **‘**age prejudice**’**:ti,ab,kw OR **‘**age stigma**’**:ti,ab,kw OR **‘**age related bias**’**:ti,ab,kw OR **‘**age related stigma**’**:ti,ab,kw OR **‘**age related discrimination**’**:ti,ab,kw OR elderspeak:ti,ab,kw OR ‘elder speak’:ti,ab,kw OR (('stigma'/exp OR 'stereotypy'/exp OR 'attitude'/de OR attitude*:ti,ab,kw OR perception*:ti,ab,kw OR perceive*:ti,ab,kw OR belief*:ti,ab,kw OR believe*:ti,ab,kw OR preconcepti*:ti,ab,kw OR stereotyp*:ti,ab,kw OR bias:ti,ab,kw OR discriminat*:ti,ab,kw OR stigma*:ti,ab,kw OR prejudic*:ti,ab,kw) **AND** ('aged'/exp OR 'geriatrics'/exp OR 'elderly care'/exp OR elder*:de,ab,ti OR eldest:de,ab,ti OR frail*:de,ab,ti OR geriatri*:de,ab,ti OR (old NEXT/1 age*):de,ab,ti OR (oldest NEXT/1 old*):de,ab,ti OR senior*:de,ab,ti OR senium:de,ab,ti OR (very NEXT/1 old*):de,ab,ti OR septuagenarian*:de,ab,ti OR octagenarian*:de,ab,ti OR octogenarian*:de,ab,ti OR nonagenarian*:de,ab,ti OR centarian*:de,ab,ti OR centenarian*:de,ab,ti OR supercentenarian*:de,ab,ti OR 'older people':de,ab,ti OR (older NEXT/1 subject*):de,ab,ti OR (older NEXT/1 patient*):de,ab,ti OR (older NEXT/1 age*):de,ab,ti OR (older NEXT/1 adult*):de,ab,ti OR 'older man':de,ab,ti OR 'older men':de,ab,ti OR 'older male*':de,ab,ti OR 'older woman':de,ab,ti OR 'older women':de,ab,ti OR 'older female*':de,ab,ti OR (older NEXT/1 population*):de,ab,ti OR (older NEXT/1 person*):de,ab,ti OR ‘ageing process*’:ti,ab,kw OR ‘aging process*’:ti,ab,kw OR ‘aged bod*’:ti,ab,kw OR ‘ageing bod*’:ti,ab,kw OR ‘aging bod*’:ti,ab,kw OR ‘ageing patient*’:ti,ab,kw OR ‘aging patient*’:ti,ab,kw OR ‘ageing people*’:ti,ab,kw OR ‘aging people*’:ti,ab,kw OR ‘ageing person*’:ti,ab,kw OR ‘aging person*’:ti,ab,kw OR ‘older individual*’:ti,ab,kw)))

4. ('attitude to aging'/exp OR (('attitude'/de OR 'perception'/de OR 'experience'/exp OR 'self concept'/de OR attitude*:ti,ab,kw OR perception*:ti,ab,kw OR perceiv*:ti,ab,kw OR experience*:ti,ab,kw) **AND** (‘personal aging*’:ti,ab,kw OR ‘own aging*’:ti,ab,kw OR ‘personal ageing*’:ti,ab,kw OR ‘own ageing*’:ti,ab,kw)) **OR** **‘**attitude toward aging**’**:ti,ab,kw OR **‘**attitudes toward aging**’**:ti,ab,kw OR **‘**attitude toward ageing**’**:ti,ab,kw OR **‘**attitude toward aging**’**:ti,ab,kw OR **‘**attitudes to ageing**’**:ti,ab,kw OR **‘**attitudes to aging**’**:ti,ab,kw OR **‘**attitudes about ageing**’**:ti,ab,kw OR **‘**attitudes about aging**’**:ti,ab,kw OR **‘**aging expectations**’**:ti,ab,kw OR **‘**expectations of ageing**’**:ti,ab,kw OR **‘**expectations of aging**’**:ti,ab,kw OR **‘**expectations about ageing**’**:ti,ab,kw OR **‘**expectations about aging**’**:ti,ab,kw OR **‘**perceptions of aging**’**:ti,ab,kw OR **‘**perceptions of ageing’:ti,ab,kw OR ‘perceptions about aging’:ti,ab,kw OR ‘perceptions about ageing’:ti,ab,kw OR ‘fear of aging’:ti,ab,kw OR ‘fear of ageing’:ti,ab,kw OR ‘aging anxiet*’:ti,ab,kw OR ‘ageing anxiet*’:ti,ab,kw)

5. (('attitude'/de OR 'perception'/de OR 'experience'/exp OR attitude*:ti,ab,kw OR perception*:ti,ab,kw OR perceiv*:ti,ab,kw OR experienc*:ti,ab,kw OR career*:ti,ab,kw OR interest*:ti,ab,kw) **AND** ('geriatrics'/exp OR geriatri*:ti,ab,kw OR 'older patient*':ti,ab,kw OR ‘ageing patient*’:ti,ab,kw OR ‘aging patient*’:ti,ab,kw OR ‘aged care’:ti,ab,kw OR 'care for the elderly':ti,ab,kw OR 'elderly care':ti,ab,kw OR eldercare:ti,ab,kw OR 'elder care':ti,ab,kw OR 'elderly patient*':ti,ab,kw OR 'elder patient*':ti,ab,kw))

*Ebsco/APA PsychINFO 25 August 2022*

1 AND (2 OR 3 OR 4) AND 5

1. DE "Medical Students" OR DE "Medical Internship" OR DE "Medical Residency" OR DE "Psychiatric Training OR TI(“medical student*” OR “medicine student*” OR intern OR interns OR internship* OR undergraduate* OR trainee* OR resident OR residents OR residenc* OR clerk OR clerks OR clerkship* OR fellow OR fellows OR fellowship* OR rotation* OR “physicians in training” OR “physician in training” OR “future physician*”) OR AB(“medical student*” OR “medicine student*” OR intern OR interns OR internship* OR undergraduate* OR trainee* OR resident OR residents OR residenc* OR clerk OR clerks OR clerkship* OR fellow OR fellows OR fellowship* OR rotation* OR “physicians in training” OR “physician in training” OR “future physician*”) OR KW((medical W0 student*) OR (medicine W0 student*) OR intern OR interns OR internship* OR undergraduate* OR trainee* OR resident OR residents OR residenc* OR clerk OR clerks OR clerkship* OR fellow OR fellows OR fellowship* OR rotation* OR (physician* W0 in W0 training) OR (future W0 physician*))

2. DE "Death Anxiety" OR DE "Death Attitudes" OR DE "Death Education" OR TI(“attitude to death” OR “attitudes to death” OR thanatophobi* OR “death anxiet*” OR “fear of death” OR “fear of dying” OR “fearing death” OR “death attitude*” OR “death avoid*” OR “death distress*” OR ((death OR mortal*) AND salience) OR “death orientation*” OR “own death” OR “own mortal*” OR ((existential*) AND (vulnerab* OR anxiet* OR suffering*)) OR “death aware*” OR “afraid of death” OR “fear of dying” OR “death thought*” OR “death acceptance” OR “accepting death” OR “denial of death” OR “facing death” OR “anxieties about death” OR “anxiety about death” OR “anxiety toward death” OR “anxieties toward death” OR “attitude toward death” OR “attitude towards death” OR “attitudes toward death” OR “attitudes towards death” OR “attitudes towards dying” OR “attitude towards dying” OR “attitudes toward dying” OR “attitude toward dying” OR “attitude to dying” OR “attitudes to dying”) OR AB(“attitude to death” OR “attitudes to death” OR thanatophobi* OR “death anxiet*” OR “fear of death” OR “fear of dying” OR “fearing death” OR “death attitude*” OR “death avoid*” OR “death distress*” OR ((death OR mortal*) AND salience) OR “death orientation*” OR “own death” OR “own mortal*” OR ((existential*) AND (vulnerab* OR anxiet* OR suffering*)) OR “death aware*” OR “afraid of death” OR “fear of dying” OR “death thought*” OR “death acceptance” OR “accepting death” OR “denial of death” OR “facing death” OR “anxieties about death” OR “anxiety about death” OR “anxiety toward death” OR “anxieties toward death” OR “attitude toward death” OR “attitude towards death” OR “attitudes toward death” OR “attitudes towards death” OR “attitudes towards dying” OR “attitude towards dying” OR “attitudes toward dying” OR “attitude toward dying” OR “attitude to dying” OR “attitudes to dying”) OR KW((attitude* W0 to W0 death) OR thanatophobi* OR (death W0 anxiet*) OR (fear W0 of W0 death) OR (fear W0 of W0 dying) OR (fearing W0 death) OR (death W0 attitude*) OR (death W0 avoid*) OR (death W0 distress*) OR ((death OR mortal*) AND salience) OR (death W0 orientation*) OR (own W0 death) OR (own W0 mortal*) OR (existential* AND (vulnerab* OR anxiet* OR suffering*)) OR (death W0 aware*) OR (afraid W0 of W0 death) OR (fear W0 of W0 dying) OR (death W0 thought*) OR (death W0 acceptance) OR (accepting W0 death) OR (denial W0 of W0 death) OR (facing W0 death) OR (anxiet* W0 about W0 death) OR (anxiet* W0 toward* W0 death) OR (attitude* W0 toward* W0 death) OR (attitude* W0 toward* W0 dying) OR (attitude* W0 to W0 dying))

**OR**

DE "Attitudes" OR DE "Attitude Formation" OR DE "Frame of Reference" OR DE "Explicit Attitudes" OR DE "Implicit Attitudes" OR DE "Student Attitudes" OR TI(attitude*) OR AB(attitude*) OR KW(attitude*)

**AND**

DE "Death and Dying" OR TI(dying OR death* OR mortal*) OR AB(dying OR death* OR mortal*) OR KW(dying OR death* OR mortal*)

3. DE "Ageism" OR DE "Age Discrimination" OR TI(ageism* OR age discriminati* OR ageist* OR agist OR “age bias*” OR “aging bias*” OR “age based discriminati*” OR “age stereotype*” OR **“**age prejudice**”** OR **“**age stigma**”** OR **“**age related bias**”** OR **“**age related stigma**”** OR **“**age related discrimination**”** OR elderspeak OR “elder speak” OR attitude* OR perception* OR perceive* OR belief* OR believe* OR preconcepti*) OR AB(ageism* OR age discriminati* OR ageist* OR agist OR “age bias*” OR “aging bias*” OR “age based discriminati*” OR “age stereotype*” OR **“**age prejudice**”** OR **“**age stigma**”** OR **“**age related bias**”** OR **“**age related stigma**”** OR **“**age related discrimination**”** OR elderspeak OR “elder speak” OR attitude* OR perception* OR perceive* OR belief* OR believe* OR preconcepti*) OR KW(ageism* OR age W0 discriminati* OR ageist* OR agist OR “age W0 bias*) OR (aging W0 bias*) OR (age W0 based W0 discriminati*) OR (age W0 stereotype*) OR (age W0 prejudice) OR (age W0 stigma) OR (age W0 related W0 bias) OR (age W0 related W0 stigma) OR (age W0 related W0 discrimination) OR elderspeak OR (elder W0 speak) OR attitude* OR perception* OR perceive* OR belief* OR believe* OR preconcepti*)

**OR**

DE "Stigma" OR DE "Stereotyped Attitudes" OR DE "Prejudice" OR DE "Cognitive Bias" OR DE "Interpretive Bias" OR DE "Social Discrimination" OR DE "Discrimination" OR DE "Social Perception" OR TI(stereotyp* OR bias OR discriminat* OR stigma* OR prejudic*) OR AB(stereotyp* OR bias OR discriminat* OR stigma* OR prejudic*) OR KW(stereotyp* OR bias OR discriminat* OR stigma* OR prejudic*)

**AND**

**[Age group]**

**OR**

DE "Geriatric Psychiatry" OR DE "Geriatrics" OR DE "Gerontology" OR DE "Elder Care" OR TI(elder* OR eldest OR frail* OR geriatri* OR (old N1 age*) OR (oldest N1 old*) OR senior* OR senium OR (very N1 old*) OR septuagenarian* OR octagenarian* OR octogenarian* OR nonagenarian* OR centarian* OR centenarian* OR supercentenarian* OR “older people” OR (older N1 subject*) OR (older N1 patient*) OR (older N1 age*) OR (older N1 adult*) OR “older man” OR “older men” OR “older male*” OR “older woman” OR “older women” OR “older female*” OR (older N1 population*) OR (older N1 person*) OR “ageing process*” OR “aging process*” OR “aged bod*” OR “ageing bod*” OR “aging bod*” OR “ageing patient*” OR “aging patient*” OR “ageing people*” OR “aging people*” OR “ageing person*” OR “aging person*” OR “older individual*”) OR AB(elder* OR eldest OR frail* OR geriatri* OR (old N1 age*) OR (oldest N1 old*) OR senior* OR senium OR (very N1 old*) OR septuagenarian* OR octagenarian* OR octogenarian* OR nonagenarian* OR centarian* OR centenarian* OR supercentenarian* OR “older people” OR (older N1 subject*) OR (older N1 patient*) OR (older N1 age*) OR (older N1 adult*) OR “older man” OR “older men” OR “older male*” OR “older woman” OR “older women” OR “older female*” OR (older N1 population*) OR (older N1 person*) OR “ageing process*” OR “aging process*” OR “aged bod*” OR “ageing bod*” OR “aging bod*” OR “ageing patient*” OR “aging patient*” OR “ageing people*” OR “aging people*” OR “ageing person*” OR “aging person*” OR “older individual*”) OR KW(elder* OR eldest OR frail* OR geriatri* OR (old N1 age*) OR (oldest N1 old*) OR senior* OR senium OR (very N1 old*) OR septuagenarian* OR octagenarian* OR octogenarian* OR nonagenarian* OR centarian* OR centenarian* OR supercentenarian* OR “older people” OR (older N1 subject*) OR (older N1 patient*) OR (older N1 age*) OR (older N1 adult*) OR (older W0 man) OR (older W0 men) OR (older W0 male*) OR (older W0 woman) OR (older W0 women) OR (older W0 female*) OR (older N1 population*) OR (older N1 person*) OR (ageing W0 process*) OR (aging W0 process*) OR (aged W0 bod*) OR (ageing W0 bod*) OR (aging W0 bod*) OR (ageing W0 patient*) OR (aging W0 patient*) OR (ageing W0 people*) OR (aging W0 people*) OR (ageing W0 person*) OR (aging W0 person*) OR (older W0 individual*))

3. DE "Aging (Attitudes Toward)"

**OR**

DE "Attitudes" OR DE "Explicit Attitudes" OR DE "Implicit Attitudes" OR DE "Student Attitudes" OR DE "Perception" OR DE "Self-Perception" OR TI(attitude* OR perception* OR perceiv* OR experience*) OR AB(attitude* OR perception* OR perceiv* OR experience*) OR KW(attitude* OR perception* OR perceiv* OR experience*)

**AND**

DE "Aging" OR DE "Cognitive Aging" OR DE "Physiological Aging" OR TI(“personal aging*” OR “own aging*” OR “personal ageing*” OR “own ageing*” OR “attitude toward aging” OR “attitudes toward aging” OR “attitude toward ageing” OR “attitude toward aging” OR “attitudes to ageing” OR “attitudes to aging” “attitudes about ageing” OR “attitudes about aging” OR “aging expectations” OR “expectations of ageing” OR “expectations of aging” OR “expectations about ageing” OR “expectations about aging” OR “perceptions of aging” OR “perceptions of ageing” OR “perceptions about aging” OR “perceptions about ageing” OR “fear of aging” OR “fear of ageing” OR “aging anxiet*” OR “ageing anxiet*”) OR AB(“personal aging*” OR “own aging*” OR “personal ageing*” OR “own ageing*” OR “attitude toward aging” OR “attitudes toward aging” OR “attitude toward ageing” OR “attitude toward aging” OR “attitudes to ageing” OR “attitudes to aging” “attitudes about ageing” OR “attitudes about aging” OR “aging expectations” OR “expectations of ageing” OR “expectations of aging” OR “expectations about ageing” OR “expectations about aging” OR “perceptions of aging” OR “perceptions of ageing” OR “perceptions about aging” OR “perceptions about ageing” OR “fear of aging” OR “fear of ageing” OR “aging anxiet*” OR “ageing anxiet*”) OR KW((personal W0 aging*) OR (own W0 aging*) OR (personal W0 ageing*) OR (own W0 ageing*) OR (attitude* W0 toward* W0 aging) OR (attitude* W0 toward* W0 ageing) OR (attitude* W0 to W0 ageing) OR (attitude* W0 about W0 ageing) OR (aging W0 expectation*) OR (expectation* W0 of W0 ageing) OR (expectation* W0 of W0 aging) OR (expectation* W0 about W0 ageing) OR (expectation* W0 about W0 aging) OR (perception* W0 of W0 aging) OR (perception* W0 of W0 ageing) OR (perception* W0 about W0 aging) OR (perception* W0 about W0 ageing) OR (fear W0 of W0 aging) OR (fear W0 of W0 ageing) OR (aging W0 anxiet*) OR (ageing W0 anxiet*))

5. DE "Aged (Attitudes Toward)"

**OR**

DE "Attitudes" OR DE "Explicit Attitudes" OR DE "Implicit Attitudes" OR DE "Student Attitudes" OR DE "Social Perception" OR DE "Perception" OR TI(attitude* OR perception* OR perceiv* OR experienc* OR career* OR interest*) OR AB(attitude* OR perception* OR perceiv* OR experienc* OR career* OR interest*) OR KW(attitude* OR perception* OR perceiv* OR experienc* OR career* OR interest*)

**AND**

DE "Geriatric Psychiatry" OR DE "Geriatrics" OR DE "Gerontology" OR DE "Elder Care" OR TI(geriatri* OR “older patient*” OR “ageing patient*” OR “aging patient*” OR “aged care” OR “care for the elderly” OR “elderly care” OR eldercare OR “elder care” OR “elderly patient*” OR “elder patient*”) OR AB(geriatri* OR “older patient*” OR “ageing patient*” OR “aging patient*” OR “aged care” OR “care for the elderly” OR “elderly care” OR eldercare OR “elder care” OR “elderly patient*” OR “elder patient*”) OR KW(geriatri* OR (older W0 patient*) OR (ageing W0 patient*) OR (aging W0 patient*) OR (aged W0 care) OR (care W0 for W0 the W0 elderly) OR (elder* W0 care) OR eldercare OR (elder* W0 patient*))

*Ebsco/ERIC 25 August 2022*

1 AND (2 OR 3 OR 4) AND 5

1. DE "Medical Students" OR DE "Student Experience" OR TI(“medical student*” OR “medicine student*” OR intern OR interns OR internship* OR undergraduate* OR trainee* OR resident OR residents OR residenc* OR clerk OR clerks OR clerkship* OR fellow OR fellows OR fellowship* OR rotation* OR “physicians in training” OR “physician in training” OR “future physician*”) OR AB(“medical student*” OR “medicine student*” OR intern OR interns OR internship* OR undergraduate* OR trainee* OR resident OR residents OR residenc* OR clerk OR clerks OR clerkship* OR fellow OR fellows OR fellowship* OR rotation* OR “physicians in training” OR “physician in training” OR “future physician*”) OR KW((medical W0 student*) OR (medicine W0 student*) OR intern OR interns OR internship* OR undergraduate* OR trainee* OR resident OR residents OR residenc* OR clerk OR clerks OR clerkship* OR fellow OR fellows OR fellowship* OR rotation* OR (physician* W0 in W0 training) OR (future W0 physician*))

2. TI(“attitude to death” OR “attitudes to death” OR thanatophobi* OR “death anxiet*” OR “fear of death” OR “fear of dying” OR “fearing death” OR “death attitude*” OR “death avoid*” OR “death distress*” OR ((death OR mortal*) AND salience) OR “death orientation*” OR “own death” OR “own mortal*” OR ((existential*) AND (vulnerab* OR anxiet* OR suffering*)) OR “death aware*” OR “afraid of death” OR “fear of dying” OR “death thought*” OR “death acceptance” OR “accepting death” OR “denial of death” OR “facing death” OR “anxieties about death” OR “anxiety about death” OR “anxiety toward death” OR “anxieties toward death” OR “attitude toward death” OR “attitude towards death” OR “attitudes toward death” OR “attitudes towards death” OR “attitudes towards dying” OR “attitude towards dying” OR “attitudes toward dying” OR “attitude toward dying” OR “attitude to dying” OR “attitudes to dying”) OR AB(“attitude to death” OR “attitudes to death” OR thanatophobi* OR “death anxiet*” OR “fear of death” OR “fear of dying” OR “fearing death” OR “death attitude*” OR “death avoid*” OR “death distress*” OR ((death OR mortal*) AND salience) OR “death orientation*” OR “own death” OR “own mortal*” OR ((existential*) AND (vulnerab* OR anxiet* OR suffering*)) OR “death aware*” OR “afraid of death” OR “fear of dying” OR “death thought*” OR “death acceptance” OR “accepting death” OR “denial of death” OR “facing death” OR “anxieties about death” OR “anxiety about death” OR “anxiety toward death” OR “anxieties toward death” OR “attitude toward death” OR “attitude towards death” OR “attitudes toward death” OR “attitudes towards death” OR “attitudes towards dying” OR “attitude towards dying” OR “attitudes toward dying” OR “attitude toward dying” OR “attitude to dying” OR “attitudes to dying”) OR KW((attitude* W0 to W0 death) OR thanatophobi* OR (death W0 anxiet*) OR (fear W0 of W0 death) OR (fear W0 of W0 dying) OR (fearing W0 death) OR (death W0 attitude*) OR (death W0 avoid*) OR (death W0 distress*) OR ((death OR mortal*) AND salience) OR (death W0 orientation*) OR (own W0 death) OR (own W0 mortal*) OR (existential* AND (vulnerab* OR anxiet* OR suffering*)) OR (death W0 aware*) OR (afraid W0 of W0 death) OR (fear W0 of W0 dying) OR (death W0 thought*) OR (death W0 acceptance) OR (accepting W0 death) OR (denial W0 of W0 death) OR (facing W0 death) OR (anxiet* W0 about W0 death) OR (anxiet* W0 toward* W0 death) OR (attitude* W0 toward* W0 death) OR (attitude* W0 toward* W0 dying) OR (attitude* W0 to W0 dying)

**OR**

DE "Student Attitudes" OR DE "Attitudes" OR TI(attitude*) OR AB(attitude*) OR KW(attitude*)

**AND**

DE "Death" OR TI(dying OR death* OR mortal*) OR AB(dying OR death* OR mortal*) OR KW(dying OR death* OR mortal*)

3. DE "Age Discrimination" OR TI(ageism* OR age discriminati* OR ageist* OR agist OR “age bias*” OR “aging bias*” OR “age based discriminati*” OR “age stereotype*” OR **“**age prejudice**”** OR **“**age stigma**”** OR **“**age related bias**”** OR **“**age related stigma**”** OR **“**age related discrimination**”** OR elderspeak OR “elder speak” OR attitude* OR perception* OR perceive* OR belief* OR believe* OR preconcepti*) OR AB(ageism* OR age discriminati* OR ageist* OR agist OR “age bias*” OR “aging bias*” OR “age based discriminati*” OR “age stereotype*” OR **“**age prejudice**”** OR **“**age stigma**”** OR **“**age related bias**”** OR **“**age related stigma**”** OR **“**age related discrimination**”** OR elderspeak OR “elder speak” OR attitude* OR perception* OR perceive* OR belief* OR believe* OR preconcepti*) OR KW(ageism* OR age W0 discriminati* OR ageist* OR agist OR “age W0 bias*) OR (aging W0 bias*) OR (age W0 based W0 discriminati*) OR (age W0 stereotype*) OR (age W0 prejudice) OR (age W0 stigma) OR (age W0 related W0 bias) OR (age W0 related W0 stigma) OR (age W0 related W0 discrimination) OR elderspeak OR (elder W0 speak) OR attitude* OR perception* OR perceive* OR belief* OR believe* OR preconcepti*)

**OR**

DE "Labeling (of Persons)" OR DE "Social Bias" OR DE "Stereotypes" OR DE "Social Discrimination" OR TI(stereotyp* OR bias OR discriminat* OR stigma* OR prejudic*) OR AB(stereotyp* OR bias OR discriminat* OR stigma* OR prejudic*) OR KW(stereotyp* OR bias OR discriminat* OR stigma* OR prejudic*)

**AND**

DE "Older Adults" OR DE "Educational Gerontology" OR DE "Geriatrics" OR DE "Gerontology" OR TI(elder* OR eldest OR frail* OR geriatri* OR (old N1 age*) OR (oldest N1 old*) OR senior* OR senium OR (very N1 old*) OR septuagenarian* OR octagenarian* OR octogenarian* OR nonagenarian* OR centarian* OR centenarian* OR supercentenarian* OR “older people” OR (older N1 subject*) OR (older N1 patient*) OR (older N1 age*) OR (older N1 adult*) OR “older man” OR “older men” OR “older male*” OR “older woman” OR “older women” OR “older female*” OR (older N1 population*) OR (older N1 person*) OR “ageing process*” OR “aging process*” OR “aged bod*” OR “ageing bod*” OR “aging bod*” OR “ageing patient*” OR “aging patient*” OR “ageing people*” OR “aging people*” OR “ageing person*” OR “aging person*” OR “older individual*”) OR AB(elder* OR eldest OR frail* OR geriatri* OR (old N1 age*) OR (oldest N1 old*) OR senior* OR senium OR (very N1 old*) OR septuagenarian* OR octagenarian* OR octogenarian* OR nonagenarian* OR centarian* OR centenarian* OR supercentenarian* OR “older people” OR (older N1 subject*) OR (older N1 patient*) OR (older N1 age*) OR (older N1 adult*) OR “older man” OR “older men” OR “older male*” OR “older woman” OR “older women” OR “older female*” OR (older N1 population*) OR (older N1 person*) OR “ageing process*” OR “aging process*” OR “aged bod*” OR “ageing bod*” OR “aging bod*” OR “ageing patient*” OR “aging patient*” OR “ageing people*” OR “aging people*” OR “ageing person*” OR “aging person*” OR “older individual*”) OR KW(elder* OR eldest OR frail* OR geriatri* OR (old N1 age*) OR (oldest N1 old*) OR senior* OR senium OR (very N1 old*) OR septuagenarian* OR octagenarian* OR octogenarian* OR nonagenarian* OR centarian* OR centenarian* OR supercentenarian* OR “older people” OR (older N1 subject*) OR (older N1 patient*) OR (older N1 age*) OR (older N1 adult*) OR (older W0 man) OR (older W0 men) OR (older W0 male*) OR (older W0 woman) OR (older W0 women) OR (older W0 female*) OR (older N1 population*) OR (older N1 person*) OR (ageing W0 process*) OR (aging W0 process*) OR (aged W0 bod*) OR (ageing W0 bod*) OR (aging W0 bod*) OR (ageing W0 patient*) OR (aging W0 patient*) OR (ageing W0 people*) OR (aging W0 people*) OR (ageing W0 person*) OR (aging W0 person*) OR (older W0 individual*))

4. **DE "Student Attitudes" OR** DE "Attitudes" OR DE "Perception" OR TI(attitude* OR perception* OR perceiv* OR experience*) OR AB(attitude* OR perception* OR perceiv* OR experience*) OR KW(attitude* OR perception* OR perceiv* OR experience*)

**AND**

DE "Aging (Individuals)" OR TI(“personal aging*” OR “own aging*” OR “personal ageing*” OR “own ageing*” OR “attitude toward aging” OR “attitudes toward aging” OR “attitude toward ageing” OR “attitude toward aging” OR “attitudes to ageing” OR “attitudes to aging” “attitudes about ageing” OR “attitudes about aging” OR “aging expectations” OR “expectations of ageing” OR “expectations of aging” OR “expectations about ageing” OR “expectations about aging” OR “perceptions of aging” OR “perceptions of ageing” OR “perceptions about aging” OR “perceptions about ageing” OR “fear of aging” OR “fear of ageing” OR “aging anxiet*” OR “ageing anxiet*”) OR AB(“personal aging*” OR “own aging*” OR “personal ageing*” OR “own ageing*” OR “attitude toward aging” OR “attitudes toward aging” OR “attitude toward ageing” OR “attitude toward aging” OR “attitudes to ageing” OR “attitudes to aging” “attitudes about ageing” OR “attitudes about aging” OR “aging expectations” OR “expectations of ageing” OR “expectations of aging” OR “expectations about ageing” OR “expectations about aging” OR “perceptions of aging” OR “perceptions of ageing” OR “perceptions about aging” OR “perceptions about ageing” OR “fear of aging” OR “fear of ageing” OR “aging anxiet*” OR “ageing anxiet*”) OR KW((personal W0 aging*) OR (own W0 aging*) OR (personal W0 ageing*) OR (own W0 ageing*) OR (attitude* W0 toward* W0 aging) OR (attitude* W0 toward* W0 ageing) OR (attitude* W0 to W0 ageing) OR (attitude* W0 about W0 ageing) OR (aging W0 expectation*) OR (expectation* W0 of W0 ageing) OR (expectation* W0 of W0 aging) OR (expectation* W0 about W0 ageing) OR (expectation* W0 about W0 aging) OR (perception* W0 of W0 aging) OR (perception* W0 of W0 ageing) OR (perception* W0 about W0 aging) OR (perception* W0 about W0 ageing) OR (fear W0 of W0 aging) OR (fear W0 of W0 ageing) OR (aging W0 anxiet*) OR (ageing W0 anxiet*))

5. **DE "Student Attitudes" OR** DE "Attitudes" OR DE "Perception" OR TI(attitude* OR perception* OR perceiv* OR experienc* OR career* OR interest*) OR AB(attitude* OR perception* OR perceiv* OR experienc* OR career* OR interest*) OR KW(attitude* OR perception* OR perceiv* OR experienc* OR career* OR interest*)

**AND**

DE "Older Adults" OR DE "Educational Gerontology" OR DE "Geriatrics" OR DE "Gerontology" OR TI(geriatri* OR “older patient*” OR “ageing patient*” OR “aging patient*” OR “aged care” OR “care for the elderly” OR “elderly care” OR eldercare OR “elder care” OR “elderly patient*” OR “elder patient*”) OR AB(geriatri* OR “older patient*” OR “ageing patient*” OR “aging patient*” OR “aged care” OR “care for the elderly” OR “elderly care” OR eldercare OR “elder care” OR “elderly patient*” OR “elder patient*”) OR KW(geriatri* OR (older W0 patient*) OR (ageing W0 patient*) OR (aging W0 patient*) OR (aged W0 care) OR (care W0 for W0 the W0 elderly) OR (elder* W0 care) OR eldercare OR (elder* W0 patient*))
