# Supplementary material for: How do ageism, death anxiety and ageing anxiety among medical students and residents affect their attitude towards medical care for older patients: a systematic review
Source: BMC Med Educ. 2024 Feb 27;24:199. doi: 10.1186/s12909-024-05147-1 (PMC10900813; doi:10.1186/s12909-024-05147-1)
Supplement: Supplementary file 2 — Additional file 2. Extraction form. [file 12909_2024_5147_MOESM2_ESM.docx]

**Appendix 2: Extraction form**

Study number: Study year: Reviewer:

Country where study was conducted

□ USA □ Other:

Authors (first 2):

Journal:

Type of paper

□ Empirical (original research)

□ Review

□ **Non-empirical** (letter, essay, opinion, conference abstract)

Language

□ English

□ Dutch

□ Other, i.e.

Underlying mechanism

□ Attitude towards older people

□ Death anxiety

□ Ageing anxiety

□ **Other, i.e.**

Population

□ Medical students

□ Residents

□ **Physicians**

□ **Other, i.e.**

Attitude towards medical care for older patients

□ Attitude towards older patients

□ Attitude towards providing medical care for older patients

□ Interest in geriatrics as a career

□ **Other, i.e.**

Include in review?

□ Yes

□ No, because

□ Uncertain, because
